# Study on the Effect of *Dalbergia pinnata* (Lour.) Prain Essential Oil on Electroencephalography upon Stimulation with Different Auditory Effects

**DOI:** 10.3390/molecules29071584

**Published:** 2024-04-02

**Authors:** Xin He, Sheng Qin, Genfa Yu, Songxing Zhang, Fengping Yi

**Affiliations:** Department of Perfume and Aroma Technology, Shanghai Institute of Technology, Shanghai 201418, China; 18018580892@163.com (X.H.); qinsheng0318@163.com (S.Q.); shyugenfa@163.com (G.Y.); zhsx@sit.edu.cn (S.Z.)

**Keywords:** *Dalbergia pinnata* (Lour.) Prain, essential oil, audio stimulation, inhalation, electroencephalograph

## Abstract

*Dalbergia pinnata* (Lour.) Prain (*D. pinnata*) is a valuable medicinal plant, and its volatile parts have a pleasant aroma. In recent years, there have been a large number of studies investigating the effect of aroma on human performance. However, the effect of the aroma of *D. pinnata* on human psychophysiological activity has not been reported. Few reports have been made about the effects of aroma and sound on human electroencephalographic (EEG) activity. This study aimed to investigate the effects of *D. pinnata* essential oil in EEG activity response to various auditory stimuli. In the EEG study, 30 healthy volunteers (15 men and 15 women) participated. The electroencephalogram changes of participants during the essential oil (EO) of *D. pinnata* inhalation under white noise, pink noise and traffic noise stimulations were recorded. EEG data from 30 electrodes placed on the scalp were analyzed according to the international 10–20 system. The EO of *D. pinnata* had various effects on the brain when subjected to different auditory stimuli. In EEG studies, delta waves increased by 20% in noiseless and white noise environments, a change that may aid sleep and relaxation. In the presence of pink noise and traffic noise, alpha and delta wave activity (frontal pole and frontal lobe) increased markedly when inhaling the EO of *D. pinnata*, a change that may help reduce anxiety. When inhaling the EO of *D. pinnata* with different auditory stimuli, women are more likely to relax and get sleepy compared to men.

## 1. Introduction

The improvement of people’s living standards has led people to pay more attention to their psychological and emotional needs, thereby raising their expectations for the acoustic environment. Studies reveal that sound and smell have a significant impact on feelings. Sound or smell stimulation is one of the variables that affect the electrical waves in the brain. Therefore emotional and psychiatric therapies have often used voice stimulation to achieve the increase in certain waves in the brain [1]. White noise and pink noise are believed to affect human psychophysiological activity significantly. White noise is noise in which the power spectral density is constant throughout the frequency domain and can be used for noise masking [2]. The abbreviation for PSD (power spectral density) is power spectra. PSD is a probabilistic statistical method that is a measure of the mean square value of a random variable. We can calculate the PSD of the EEG signal to describe the distribution of the power of the random signal along the frequency. It is one of the potential sounds that can be heard by the human ear between 20 and 20,000 Hz and can be used to improve cognitive performance [3] and promote sleep [4]. It is a mixture of all audible sound frequencies that produce a “buzzing” sound, similar to radio static, a hair dryer, and television static. We often hear white noises in our daily lives, such as the sound of rain, the sound of ocean waves, and the sound of the wind blowing leaves. Pink noise is randomly distributed in low frequency bands and is a signal or process with a power spectral density (energy or power per hertz) that is inversely proportional to frequency in a characteristic spectrum [5]. In pink noise, there is an equal amount of noise power in each octave. Studies have shown that it has a significant effect on improving sleep quality [6] and memory [7]. Traffic noise is one of the most prevalent noises in our daily lives. It refers to the sound that hinders people’s normal life generated by various means of transportation while driving, such as the whistle of ships, the roar of airplanes, the sound of car motors, the sound of tire rubbing, etc. When traffic noise exceeds a certain limit, it can seriously damage work efficiency and physical health, resulting in hearing impairment [8], anxiety [9], sleep disorders [10,11], cardiovascular diseases [12], and learning disabilities [13,14]. Its negative effects include both short-term and long-term effects, with annoyance being one of its most important outcomes [15,16]. Annoyance refers to negative emotions such as unpleasantness, dissatisfaction, and restlessness, which ultimately manifest as anxiety and stress. With the improvement of people’s living standards, people pay more attention to their psychological and emotional needs.

Smell can affect a person’s mood [17] and alter cognitive performance [18]. Essential oils are volatile liquids extracted from plants that produce a characteristic odor. Aroma inhalation is a fast, convenient, and safe method [19]. Inhaling these essential oils to treat mental and physical balance can relieve stress and rejuvenate the person for the next day’s work [20]. The olfactory nerve, which runs from the nose to the brain, is the site of action for these essential oils. In addition, the olfactory nerve is the only cranial nerve that is directly exposed to external stimuli, and they project directly into the cerebral cortex, resulting in intense stimulation [21]. During aroma inhalation, natural essential oils volatilize into the air, affecting the limbic system and hypothalamus through the olfactory nerve, which has a calming effect on the nervous and endocrine systems and controls physiological and psychological changes. The delivered aroma particles lead to the production of neurotransmitters such as dopamine and serotonin, which lead to states of sedation, relaxation, stimulation, and excitement [22]. There is an increasing trend towards the use of this therapy for the treatment of sleep disorders [23,24]. They provide people with a sense of well-being in a way that supports the body [25].

Natural and artificial sounds are thought to significantly affect human cognitive abilities. Nature sounds include the sounds of living organisms, such as bird chirping, rain, running water, and ocean waves [26]. At the same time, artificial or artificial sounds refer to the sounds of similar objects in everyday life, such as the sound of an electric car engine, a person’s screams, the sound of a fake camera shutter, the sound of a piano playing, the ringing of a telephone, and the ticking of a clock. In general, sounds can be divided into pleasant and unpleasant sounds. A pleasant sound is a sound that is good, pleasant, or attractive, such as music, singing, and birdsong. However, an unpleasant sound is a sound that has an unpleasant effect on the ears and is irritating when heard, such as traffic noise. Both music and noise can have a significant impact on cognitive function, although the nature of these effects depends on the type and intensity of sound, as well as an individual’s personal preferences and characteristics [27,28]. Some studies have shown that sound can enhance the brain’s cognitive abilities. Sayed Daud et al. [29] studied the impact of auditory white noise on the memory performance of 60 college students using different difficulty levels in visual object-number pair assessment. The results of the study suggest that listening to auditory white noise while memorizing and learning visual items can improve memory performance. Zhou et al. [6] selected subjects for nighttime sleep experiments in a pink noise environment. Stable pink noise has a significant effect on reducing brain wave complexity, inducing more stable sleep time, and improving individual sleep quality. Gilani et al. [30] performed field measurements to validate the application of standard noise models, which were later used to present the acoustic environment. Through a questionnaire survey of residents, the impact of exposure in the form of annoyance and the level of consciousness were assessed. Poor sleep quality, sensitivity to noise, and perceived noise in the home are significant risk factors for annoyance.

Today, the use of alternative and aroma therapies with mainstream medicine has gained momentum. Aromatherapy is a complementary treatment that uses essential oils as the main therapeutic agent to treat a variety of ailments. Inhalation and topical use of these essential oils to treat mental and physical balance is the foundation of aromatherapy. The therapy of these essential oils is known to relieve stress and rejuvenate for the next day’s work. The olfactory nerve, which runs from the nose to the brain, is the site of action for these essential oils. When reviewing the literature, we found that a large number of studies have been conducted to study the effects of this therapy on the human brain and its mood. Its role in mood, alertness, and mental stress in healthy subjects has received much attention. Some researchers have attempted to study the effects on brain capacity, reaction time, and some spontaneous behaviors through EEG patterns and functional imaging studies [31]. Electroencephalogram (EEG) is a suitable method for measuring central nervous system activity, particularly in the cerebral cortex. Awake brain activity can be identified by the frequency recorded during brain functions, such as excitement, anxiety, and sedation [32]. EEG (electroencephalogram) is defined as the scalp indicating the electrical activity produced by recorded neurons in the brain. EEG can be categorized into two groups according to the endogeneity of recorded activity. One group is resting-state EEG, which refers to endogenous or intrinsic neural activity without a specific stimulus or task imposed; the other is task-related EEG, which is induced or evoked by an exogenously imposed stimulus or task [33]. Li et al. [34] reviewed the widely used resting-state EEG signal processing techniques. They examined in detail spectral, connectivity, and microstate analysis, covering the oft-used EEG measures, practical issues involved, and data visualization. Zhang et al. [35] reviewed the widely used task-related EEG signal processing techniques. They discussed the methods to extract and visualize event-related potentials in the time domain and event-related oscillatory responses in the time-frequency domain. Readers can feel the electrical activity of the brain more intuitively through EEG. EEG measures and amplifies the electrical activity of the scalp, which is the result of neuronal activity in the cerebral cortex, and is a relatively objective and noninvasive method that allows for continuous measurement [36]. Typically, EEG signals include delta waves (0–4 Hz), theta waves (4–8 Hz), alpha waves (8–13 Hz), and beta waves (13–30 Hz). Different brain waves reflect different effects on the brain [37,38]. Table 1 below describes the cerebral rhythms in detail.

The scientific literature confirms that EEG signals are influenced by sounds heard and various odors inhaled [40,41]. Some scent beliefs and claims have been reported to be valid, but have not been scientifically proven, or there are limited studies whose results cannot be generalized to larger groups. Among them, the aroma of the essential oil of *D. pinnata* is of natural plant origin. Essential oil is a volatile liquid extracted from the fragrance of *D. pinnata*, which has the characteristics of long-lasting fragrance and slow volatilization. The effects of essential oil on the human body and mind, regardless of the method used to study its effect on electroencephalogram, have so far only been a small number of studies. In addition, when sound is introduced, odor can further affect people’s mood [42]. However, there is little research on the effects of sound on the brain after inhaling aromas.

Therefore, this study aimed to investigate the effects of different auditory stimuli on the EEG signal of the inhalation of essential oil. White noise, pink noise and traffic noise were selected as auditory variables, and the EEG changes of subjects inhaling essential oil under noise stimulation were recorded. In this study, we hypothesized that different auditory stimuli would lead to distinct EEG patterns during the inhalation of essential oil. When stimulated by white and pink noises, essential oil may have a significant effect on improving sleep stability. Stimulated by traffic noise, essential oil may have a significant effect on improving mood. The EEG power spectra of various microscopic states induced by the inhalation of essential oils were analyzed by using the fast Fourier transform analysis method. This study uses essential oil to study the synergistic effects of smell and hearing, providing a new way to study the effects of essential oils, supporting both naturopathic and complementary medicine.

## 2. Results

### 2.1. Volatile Components of Essential Oil

The EO obtained from *D. pinnata* was pale brown in color with a balsamic, herbal and medicinal aroma. Based on the GC-MS results, the Kovats index was used to calculate the retention index based on n-alkanes. The volatile components of essential oils were determined from Table 2, with a total of 21 compounds, including ethers (84.95%), phenols (11.19%), terpenes (2.30%), ketones (0.51%), alcohols (0.34%) and aldehydes (0.17%).

### 2.2. Comparison of Spectrograms before and after Inhalation of EO

**Figure 1:**
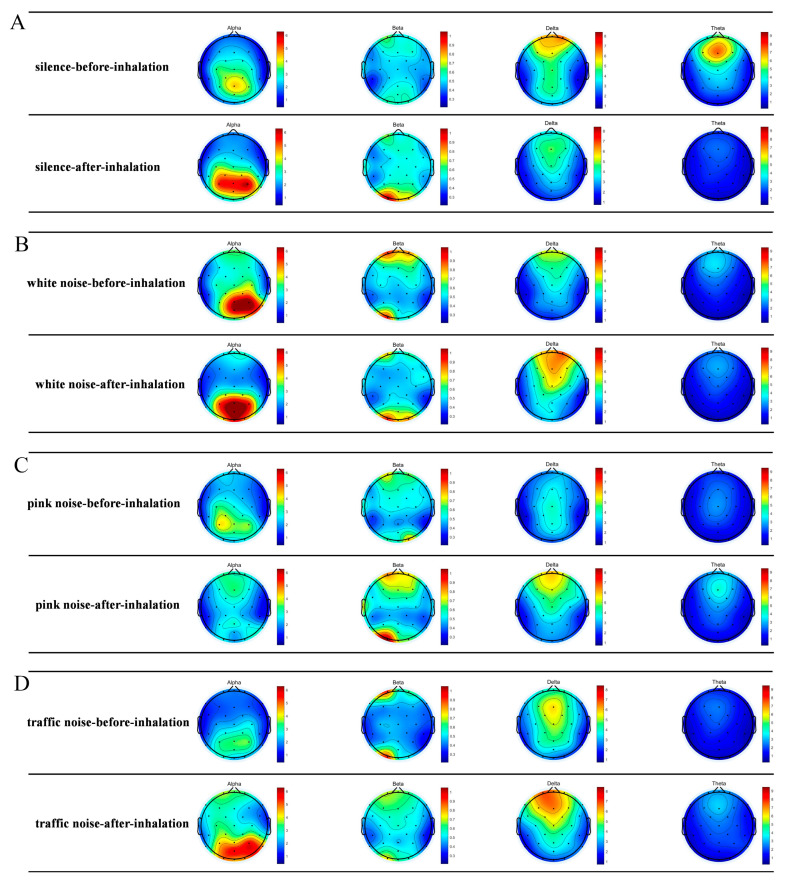
EEG spectra before and after inhalation of essential oils. Using the FFT in EEGLAB v2023.0 software (University of California, San Diego, available form http://www.sccn.ucsd.edu/eeglab/, accessed on 25 June 2023), the brain activity of 30 subjects in the same microscopic state was summarized. (**A**) Silent environment; (**B**) White noise; (**C**) Pink noise; (**D**) Traffic noise. When essential oils are inhaled, there are significant changes in each area.

The EEG vividly illustrates the brain activity in different microscopic states before and after the subjects inhaled the essential oils. Delta waves and theta waves mainly occurred in the Fp and F regions, beta waves mainly occurred in the Fp and O regions, and alpha waves mainly occurred in the P and O regions. When inhaling essential oils, the delta and theta waves in the silent group tended to weaken slightly, and the alpha waves tended to increase significantly. The delta waves of the white noise group and the pink noise group showed a significant strengthening trend. The beta waves of the traffic noise group were significantly weakened, and the alpha and delta waves had a significant strengthening trend, as shown in Figure 1.

### 2.3. Variations in the Each Electrode Point

The electrode cap defines the group of electrodes responsible for each brain region. Electrodes are used to study the activity of each area of the brain. Based on the distribution of delta, theta, alpha and beta in the spectrum, the Fp, F, P and O regions of the brain were selected for analysis.

**Figure 2:**
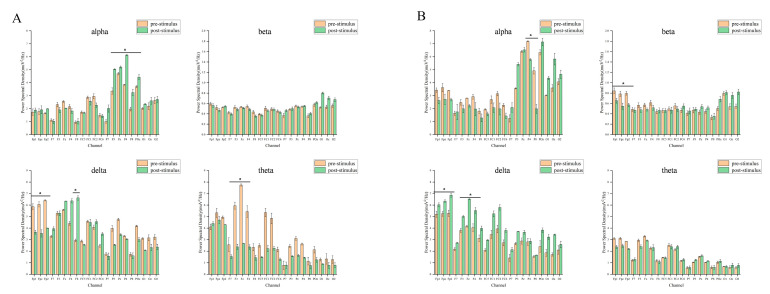

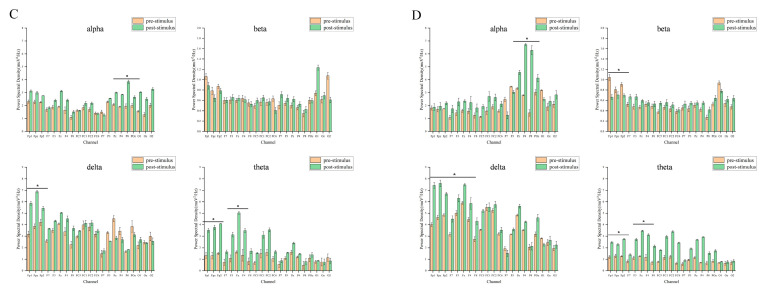
Comparison chart of each group of single-channel analyses. (**A**) Silent environment; (**B**) White noise; (**C**) Pink noise; (**D**) Traffic noise (*p*-values: * < 0.05).

A single-channel analysis was used to create a histogram of essential oil before and after stimulation. As can be seen from Figure 2, the alpha waves in the silent olfactory group mainly appeared in the posterior brain region, with obvious changes in the P area, and a significant increase in P4 compared with that before essential oil stimulation. There was no significant change in the beta waves. Delta waves showed significant changes in the Fp and F zones. Theta waves showed significant changes in the F region, with F3, Fz, and F4 attenuated by about 60% compared to those before essential oil stimulation. The beta waves of the white noise group were significantly weakened in the Fp region. The alpha waves occurred mainly in the hind brain region, and F4 and F8 were significantly increased by about 30% after stimulation with essential oil. The delta waves showed a significant increase after being stimulated by essential oil. Theta waves occurred mainly in the forebrain region and were slightly attenuated compared to before essential oil stimulation. The alpha waves of the pink noise group increased significantly after being stimulated by essential oil. There was no significant change in the beta waves. After the delta waves were stimulated by essential oil, the Fp zone increased significantly, increasing by about 60%. Theta waves appeared mainly in the forebrain region, and after stimulation with essential oil, the Fp and F areas increased by about 180%. The alpha waves of traffic noise mainly appeared in the hindbrain area, and there were obvious changes in the P area, and the P4 and P8 increased by about 150% compared with those before essential oil stimulation. Beta waves were significantly weakened in the Fp region after stimulation by essential oil. Delta waves were significantly increased in the Fp and F zones after stimulation with essential oils. Theta waves increased significantly after stimulation by essential oil.

### 2.4. Comparison of Energy before and after Inhalation of EO

A regional comparison plot of the EO/no EO group was created using a single-channel analysis. The Fp, F, P, and O regions of the brain were selected for analysis and comparison. 

**Figure 3:**
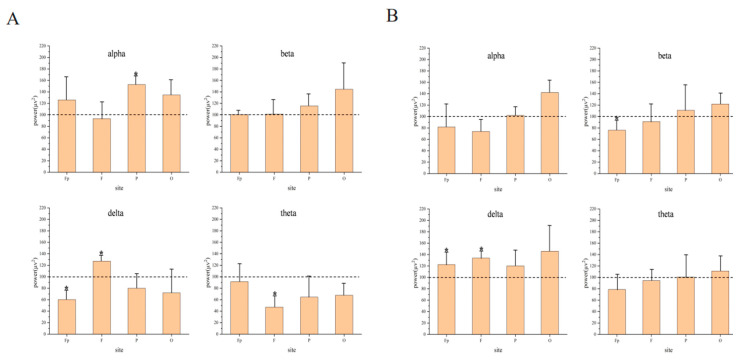

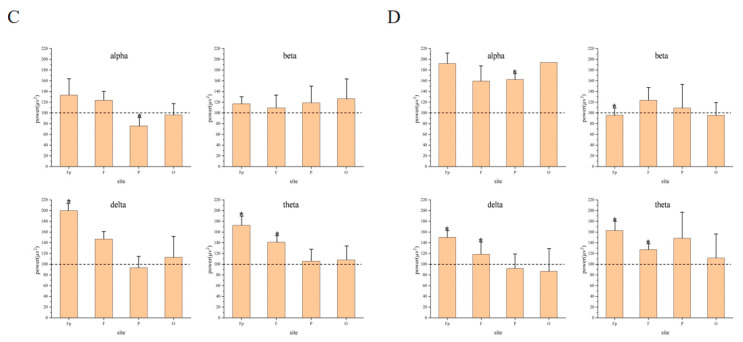
Effect of essential oils on Fp, F, P, and O regions under different auditory stimuli. (**A**) Silent environment; (**B**) White noise; (**C**) Pink noise; (**D**) Traffic noise (*p*-values: * < 0.05).

According to the energy results of the brain functional area shown in Figure 3, the energy of the theta waves in the silent olfactory absorption group decreased overall. The P and O regions of the alpha and beta waves increased significantly, and the energy of the P region of the alpha waves increased by about 150%. The delta waves of the F zone had a significant increase of about 130%. The energy of the Fp, F, and O regions of the delta waves was significantly weakened, and the Fp region was weakened by about 60%. The energy of the delta waves in the white noise sniffing group increased overall. The Fp region of the beta waves was significantly weakened by about 75%. There was no significant change in alpha and theta waves, as shown in Figure 3B. The energy of the beta waves in the pink noise sniffing group increased slightly overall.

The energy of the Fp region and the F-region of the alpha, delta and theta waves significantly increased, and the energy of the Fp region of the delta waves significantly increased by about 200%, as shown in Figure 3C. The energy of the alpha waves of the traffic noise group increased significantly. The Fp region of the beta waves weakened slightly. There was a significant increase in both Fp and F region energies of both delta and theta waves compared to before essential oil stimulation, as shown in Figure 3D.

### 2.5. The Effect of Sex Differences on Brainwave Energy

There is a significant gender difference in brainwave energy during EO inhalation. However, the energy of both delta and theta waves in men was significantly lower in silent environments compared to women. In females, theta waves were significantly increased in the Fp and F regions in the pink noise environment. In males, there is a significant increase in the energy of alpha waves in the environment of traffic noise. There was no significant difference in EEG activity between men and women in the white noise environment after EO inhalation, as shown in Table 3.

## 3. Discussion

*D. pinnata* is a traditional medicinal plant in China, which is widely used as a spice and ethnic medicinal plant resource in the distribution area for its pungent taste and warmth. It is known to return to the liver and the spleen meridian, to regulate qi and to relieve pain [43]. The essential oil obtained from *D. pinnata* was a reddish-brown liquid with a balsamic, herbal and medicinal aroma. A total of 21 compounds were identified by gas chromatography-mass spectrometry. In this study, the most abundant components in essential oils were ethers (84.95%) and phenols (11.19%). Elemicin has a strong hypnotic effect, is a mosquito repellent and insecticidal, and exhibits antifungal, anti-pneumonia and other effects. Methyl eugenol has antihypertensive, antioxidant, and antibacterial effects. Elemicin is mainly used for medical anesthesia. Methyl eugenol can penetrate the blood–brain barrier and enter the brain tissue to exert central inhibition and is used for the anesthesia of rodents [44]. Inhaling the *D. pinnata* essential oil can make people relaxed and calm under auditory stimulation. This may be due to the inhibition of nerves in the brain by elemicin and methyl eugenol in essential oil components. Zhou et al. [45] used supercritical CO_2_ extraction to extract essential oil, and a total of 14 volatile components were identified by gas chromatography-mass spectrometry, the main components of which were elemicin (91.06%) and methyl eugenol (3.69%). Previous studies have clearly shown that essential oils have pharmacological effects, such as antioxidant, antimicrobial, anti-inflammatory, and antidepressant effects [41,46,47]. 

In recent decades, many studies have shown that the aroma of essential oils and their individual components exhibit a variety of positive psychophysiological functions by altering brainwave activity in humans [40,48]. In addition, these studies clearly show that EEG is a suitable technique to understand the effects of scent on brainwave activity. In this study, inhalation of essential oils significantly increased the parietal region of alpha waves and the frontal region of theta waves. The inhalation of essential oil with different auditory stimuli had a significant effect on EEG signals, and there were obvious gender differences.

According to the EEG spectrum, different modes of power spectral density increase and decrease were generated in the Fp, F, P and O regions. To better understand the effects of stimulation, we used single-channel analysis to create a comparison plot of brain rhythms (alpha, beta, delta, and theta) before and after essential oil stimulation. As shown in Figure 2, the alpha and theta waves were significantly affected by the stimulation of essential oils in a silent environment. In the pink noise and traffic noise environments, the power spectral density of alpha waves and theta waves increased significantly. The theta waves of auditory pink noise increased by about 180% in the Fp and F regions. The overall theta waves of auditory traffic noise increased significantly by about 100%. Since the rhythm of alpha waves and theta waves reflects a relaxed state of mind, it can indicate that the subject is affected by auditory pink noise and auditory traffic noise. Theta waves are mainly found during deep meditation and are also found in the hippocampus and cortical regions. Theta waves have been implicated in the subconscious, which controls sleep, drowsiness, imaginative thinking, and creativity [49]. A decrease in theta waves activity is associated with the formation of memories. In addition, theta waves are thought to maintain attention during difficult tasks [50]. Significant changes in power spectral density due to inhalation of essential oils may be related to drowsiness or meditative states in the brain. The alpha waves of auditory pink noise and auditory traffic noise were significantly increased in the P area. It can be seen that the psychological and cognitive state of the subjects stimulated by the essential oils in pink noise and traffic noise is relaxed and calm.

When conducting attention-based assessments, beta wave rhythms typically reflect an individual’s level of attention, alertness, and arousal. The higher the beta waves’ energy, the higher the level of cognition of the stimulus. The beta waves’ energy of the auditory white noise in Figure 3 is significantly reduced in the Fp region. The beta waves’ energy of silent and audible pink noise is slightly increased, while the beta waves’ energy of traffic noise is slightly reduced. It can be seen that the subject’s cognitive state is awake when stimulated by essential oils in silent and pink noise. Subjects are more comfortable in white noise environments than in silent environments. However, excessive beta waves can also cause anxiety [51,52]. When the subject is in an auditory traffic noise environment, there is a significant increase in beta waves, as shown in Figure 1. It can be seen that the traffic noise environment may cause discomfort and tension in the subject. In a traffic environment, essential oils stimulate the mind for relaxation and calming. The delta waves are associated with deep relaxation and sleep in our lives. As shown in Figure 3, the delta waves increase by about 20% when stimulated by essential oils in a white noise environment. It can be seen that, in the environment of auditory white noise, the reduction essence has a drowsy and relaxing effect on the body but has no obvious effect on improving concentration.

Sex differences and reproductive hormones have a strong influence on human odor perception [53]. Corsi Cabrera et al. [54] found that sex differences affect EEG activity during cognitive function. The authors report that males exhibit significantly higher relative beta activity. The total number of cells was 16.2 million in females, and 9.2 million in males, a significant difference of 43.2%. The number of neurons in females reached 6.9 million, being no more than 3.5 million in males, a difference of 49.3%. The number of non-neuronal cells also proved higher in women than in men: 9.3 million and 5.7 million, respectively, a significant difference of 38.7%. Oliveira Pinto et al. [55] showed that there was a significant gender difference in the absolute number of total neuronal and non-neuronal cells, favoring women by about 40%. These differences in quantitative cellularity may have a functional impact. In many species, females have a better sense of smell than males, and this increased sensitivity may be based on biological significance [56]. In addition, the large nerve fibers that connect the two cerebral hemispheres are larger in females than males [57]. A larger corpus callosum allows for freer communication between the hemispheres, so female neurons produce more synapses [58]. In this study, there were significant gender differences before and after inhalation of essential oils under different auditory stimuli. Compared with before essential oil stimulation, the theta waves in women in the pink noise environment were significantly increased in the Fp and F regions. The energy of the alpha waves in males increased significantly in the environment of traffic noise. It can be seen that women are more likely to relax and be sleepy when stimulated by essential oils.

The data from this study clearly show that the drop in different auditory stimuli can effectively stimulate brain wave activity in different areas of the brain. In addition, the main components in essential oils, elemicin and methyl eugenol, may play a key role in the odor profile of essential oils, resulting in significant changes in EEG activity. Some studies have shown that music can enhance brain plasticity, the brain’s ability to change and adapt to new experiences. Later experiments can use music as an auditory stimulus. Different concentrations of essential oils have different effects on a person’s mood and brain waves. One can study the dose-dependent effects of essential oils. Our research on sex differences in EEG is relatively superficial. In subsequent studies, the underlying mechanisms of sex differences in EEG activity can be investigated.

## 4. Materials and Methods

### 4.1. GC-MS Analysis of EO

*D. pinnata* was purchased from Zhejiang Yifeng Cosmetics Co., Ltd. (Shaoxing, China). The essential oil was extracted from *D. pinnata* using the supercritical carbon dioxide extraction method and stored at 4 °C until use. We used supercritical fluid extractor (HA220-50-06, Nantong Huaan Supercritical Extraction Co., Ltd., Nantong, China) to extract essential oil from *D. pinnata*. Under the conditions of pressure (35 MPa) and temperature (75 °C), carbon dioxide flow rate of 10 mL/min, and 60 min of extraction, the essential oil was extracted. The samples were kept in the Fragrance and Flavor Laboratory of Shanghai Institute of Technology. In this experiment, a concentration of 100% *D. pinnata* essential oil was used.

Agilent GC-6890 coupled with MS-5973 (Santa Clara, CA, USA) was used for gas chromatography-mass spectrometry analysis of essential oils. The column was HP-INNOWax (260 °C: 60 m× 0.25 mm × 0.25 μm; Agilent, Santa Clara, CA, USA). The GC was operated under temperature programming conditions with an initial temperature of 40 °C and maintained for 6 min. First hold at 3 °C/min to 100 °C for 0 min, then 5 °C/min to 230 °C for 10 min. Samples were analyzed in unsegmented mode. The carrier gas was helium, the flow rate was 2 mL/min, and the mass spectrometry ionization method was electron shock (70 eV). The results were matched with the NIST (National Institute for Standards and Technology) database to determine the essential oil components, and the area normalization method was used to calculate the relative content of each component of the essential oils.

The retention indexes of each composition relative to n-alkanes (C8–C20) under the same operating conditions were calculated using Kovats indexes.

### 4.2. Preparation of Sound and Smell

Each participant was asked to listen to white, pink, and traffic noise, and to smell essential oils during EEG data collection. Auditory stimulation is presented binaurally to each participant at a comfortable auditory level through plugged-in headphones. According to the assessment of the decibel metering software, these audios are played at a volume of 40–55 dB to minimize harmful effects on the participant’s auditory perception. Olfactory stimulation provides aroma to each participant through diffuser. Auditory stimulation procedures were created using CapCut v2.3.0 software (Shenzhen Myotee Science and Technology, Shenzhen, China, available form https://www.capcut.cn/web, accessed on 28 June 2023) to standardize the experimental time for each participant.

### 4.3. Subjects

This study studied 30 university participants (15 males and 15 females), aged between 20 and 25 years. We recruited participants by posting announcements on campus. Each participant was paid RMB 200. All participants had no history of psychiatric illness (e.g., epilepsy, learning disabilities, acute anxiety, depression and migraine), no history of psychotropic drug use, no history of respiratory illness, no history of allergy to incense, and finally no particularly pleasant or unpleasant memories of the aroma of essential oils. In addition, after the participants were briefed on the purpose and nature of the study, each participant received informed written consent and was paid monetarily. This study follows the guiding principles of the Declaration of Helsinki and the Tokyo Declaration on human beings and was approved by the Ethics Committee of Shanghai Jiao Tong University with the approval number #B2021153I, and informed consent was obtained.

### 4.4. Experimental Design

**Figure 4:**
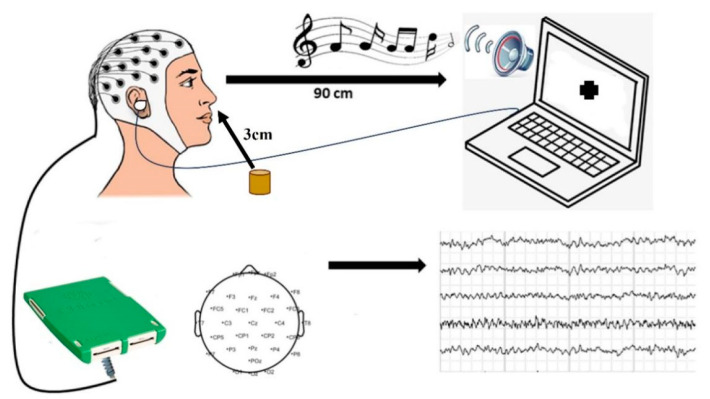
Description of the data-collection process.

The temperature of the experimental site was precisely controlled at 26 °C and the humidity was 70%. After entering the lab, subjects were asked to wash their hair with unscented shampoo and blow dry their hair. The subject wore the electrode cap appropriately, and then injected conductive glue into the electrode channel to reduce the resistance to less than 5 KΩ. An undiluted essential oil (0.10 g) was added to the diffuser and placed approximately 3 cm in front of the subject’s nose to ensure that the aroma could be smelled. The diffuser used for aroma delivery was purchased from Dr. Wong Lab (Shenzhen, China). Noise intervention is achieved through in-ear headphones. During the experiment, participants stared at the black cross on a white screen to keep their attention, as shown in Figure 4.

**Figure 5:**
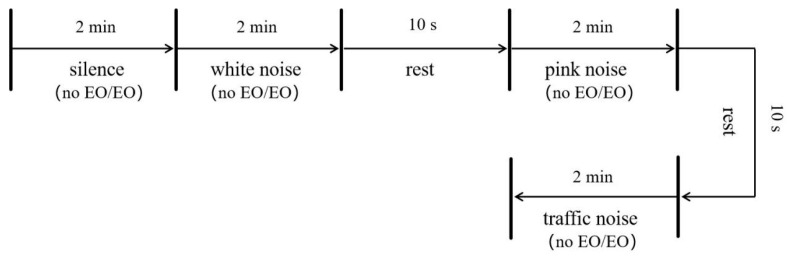
Experimental time design.

The experiment began with no audio, and the participants were then exposed to three types of audio. Participants were also asked to sit in comfortable chairs and focus on blank recordings (silent, white noise, pink noise, and traffic noise, no EO) and olfactory stimuli (no sound, white noise, pink noise, and traffic noise, with EO) for experimental times, as shown in Figure 5.

### 4.5. EEG Signal Acquisition

EEG recordings (eggoTM mylab, ANT Neuro, Hengelo, Netherlands) were recorded using a 32-electrode system. These recordings include the prefrontal polar (Fp), frontal lobe (F), central zone (C), parietal lobe (P), occipital lobe (O), and temporal lobe (T) regions. Figure 4 shows the electrode distribution, with reference electrodes M1 and M2 removed. The EEG sampling rate of the measured object was 500 Hz, and the data were filtered in the range of 1~40 Hz.

The value of each signal is determined by the sampling rate and duration. Due to the periodicity of the 30 channel and 30 subject electrode position signals, the signal was split into 2 s intervals at a sampling rate of 500 Hz during the test, and the fragment length L = 500 × 2 s. FFT (fast Fourier transform) is a fast algorithm of discrete Fourier transform, which is obtained by improving the algorithm of discrete Fourier transform according to the characteristics of discrete Fourier transform, such as odd, even, virtual and real. FFT was performed for all time points in the original EEG signal. Delta, theta, alpha, and beta waves were extracted from four channels [59]. The intensity of brainwave activity can be described by spectral and single-channel analysis. The energy of brain waves is used to quantify the results [60].

### 4.6. Statistical Analysis

All data sets that passed the normality test were analyzed using one-way analysis of variance (ANOVA) to determine the effect of the various treatment factors. Multiple comparisons were performed using the least significant difference (LSD) method to determine statistically significant differences (*p* < 0.05) [60].

## 5. Conclusions

The GC-MS analysis revealed that the EO of *D. pinnata* was mainly composed of ethers (84.95%) and phenols (11.19%). In addition, EEG power spectrum values changed significantly when the EO of *D. pinnata* was inhaled. In a silent and white noise environment, inhalation of the EO of *D. pinnata* can make people relax and enhance delta waves by 20%. In pink noise and traffic noise, inhaling the EO of *D. pinnata* can reduce anxiety and make people relaxed and calm. Sex differences also have a strong influence on EEG activity when inhaling aroma. Under the stimulation of essential oils, women’s theta wave energy increased significantly. Women are more likely to relax and get sleepy compared to men.

## Figures and Tables

**Table 1:** Classification of brain waves and their properties [39].

Brain Rhythms	Amplitude (μV)	Frequency (Hz)	Brain States
Delta	100–200	0–4	Deep sleep or unconscious state
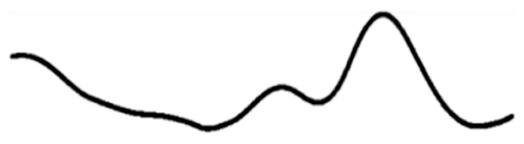			
Theta	5–10	4–8	Drowsiness
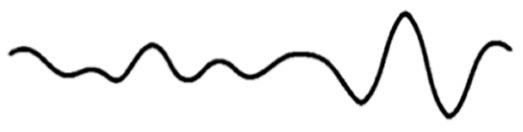			
Alpha	20–80	8–13	Relaxed awareness without attention
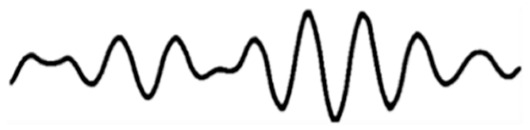			
Beta	1–5	13–30	Active thinking
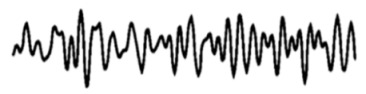			

**Table 2:** Components of *D. pinnata* essential oil as determined via GC-MS.

No.	Compound	RI ^a^	RI ^b^	Area%
1	Linolool	1548	1559	0.12
2	(+/−)-p-Menthan-3-ol	1637	1645	0.04
3	γ-Selinene	1699	1691	0.06
4	beta-cubebene	1703	1543	0.06
5	(+)-Car-3-ene	1802	1748	0.19
6	1-methyl-4-[(1*R*)-1,2,2-trimethylcyclopentyl]benzene	1861	1811	0.03
7	(−)-Calamenene	1890	1849	0.01
8	trans-5-Butyl-4-methyldihydro-2(3H)-furanone	1894	1865	0.40
9	1,2-dimethoxy-4-propylbenzene	1948	1897	1.49
10	Whiskey lactone	1980	1968	0.24
11	Methyl eugenol	1998	1985	11.19
12	Cinnamaldehyde	2044	2043	0.04
13	cis-Nothosmyrnol	2060	2071	0.02
14	Cedrol	2086	2149	0.15
15	(+)-Cyclosativene	2425	2492	0.06
16	isomethyleugenol	2639	2594	0.30
17	Elemicin	2657	2645	84.10
18	(+)-γ-Maaliene	2714	2691	0.40
19	Isoelemicin	2992	2803	0.84
20	Acetoveratrone	3041	2975	0.11
21	3,4,5-Trimethoxybenzaldehyde	3061	3039	0.13

^a^ Retention index calculated from the Kovats index. ^b^ Retention index for the chemical composition from the NIST library.

**Table 3:** Significance analysis results of essential oils on Fp, F, P, and O regions in women and men under different auditory stimuli.

Site	Noise	Wave	After Inhalation/Before Inhalation (%)	
			Girl	Boy
Fp	silence	alpha	91.14	147.80
		beta	97.66	100.76
		delta	102.16	37.69 *
		theta	131.13	24.56
	white noise	alpha	103.59	57.13
		beta	69.77 *	88.50 *
		delta	119.36	129.00 *
		theta	89.90	69.30
	pink noise	alpha	144.03	123.44
		beta	125.76	102.74
		delta	170.83 *	209.03 *
		theta	217.92 *	115.61
	traffic noise	alpha	81.09	215.71
		beta	83.27 *	115.51
		delta	137.53 *	169.51 *
		theta	163.24 *	162.00 *
F	silence	alpha	89.27	96.54
		beta	101.57	101.93
		delta	139.62 *	64.69 *
		theta	150.57 *	24.59 *
	white noise	alpha	88.77	60.87
		beta	92.47	89.50
		delta	122.10	149.17 *
		theta	112.39	81.76
	pink noise	alpha	115.97	130.53
		beta	116.22	100.33
		delta	127.48	171.87
		theta	208.59 *	104.09
	traffic noise	alpha	127.04	217.77
		beta	119.58	128.24
		delta	98.04	118.83 *
		theta	172.25 *	94.01
P	silence	alpha	217.48 *	96.02
		beta	122.52	113.28
		delta	89.88	63.91
		theta	138.91 *	41.36 *
	white noise	alpha	117.28	74.07
		beta	106.72	117.60
		delta	132.36	106.61
		theta	105.69	95.97
	pink noise	alpha	107.73	52.41 *
		beta	123.27	112.44
		delta	108.65	77.90
		theta	133.18	88.20
	traffic noise	alpha	165.61 *	158.50 *
		beta	118.35	97.90
		delta	102.16	78.50
		theta	218.12	104.67
O	silence	alpha	214.71	74.58
		beta	155.16	137.61
		delta	69.87	57.40
		theta	96.33	45.56
	white noise	alpha	164.66	109.20
		beta	95.48	168.56
		delta	147.16	143.45
		theta	114.35	107.23
	pink noise	alpha	129.46	66.17
		beta	115.01	143.37
		delta	132.54	86.99
		theta	99.92	118.23
	traffic noise	alpha	100.90	105.65
		beta	105.94	84.99
		delta	96.60	74.85
		theta	129.63	98.81

*p*-values: * < 0.05.

## Data Availability

The presented data are available upon reasonable request from the corresponding author.
